# The expert’s fixation: cognitive efficiency and attentional selectivity in wheelchair curling athletes across the expertise spectrum

**DOI:** 10.3389/fpsyg.2026.1739477

**Published:** 2026-02-20

**Authors:** Wanting Li, Xiaokun Zhang, Chunzhou Zhao, Jianrui Li, Bo Li

**Affiliations:** 1 Harbin Sport University, Harbin, China; 2 College of Physical Education Science, Harbin Normal University, Harbin, China; 3 College of Physical Education, Chizhou University, Chizhou, China

**Keywords:** cognitive efficiency, eye-tracking, Paralympic sport, quiet eye, visual attention, wheelchair curling

## Abstract

**Background:**

In precision Paralympic sports like wheelchair curling, visual attention is a paramount cognitive determinant of performance. While expertise-dependent differences in gaze behavior are documented in various sports, the specific cognitive mechanisms—particularly attentional selectivity and efficiency—underpinning elite performance in wheelchair curling remain unexplored. This study investigated the visual attention patterns of disabled curling athletes across skill levels to uncover the cognitive strategies associated with expertise.

**Methods:**

Forty-eight disabled curling athletes were stratified into elite (Paralympic/World Champions, *n* = 16), general (National Champions, *n* = 16), and novice (Provincial Champions, *n* = 16) groups. Using Tobii Pro Glasses 3, eye movements were recorded during stone deliveries. Metrics included the number of fixations, fixation duration, and their distribution across predefined Areas of Interest (AOIs: Release Zone, Stone Path, Target/House, Other Areas). Performance was quantitatively assessed by international-level judges based on stone placement accuracy.

**Results:**

A significant Group × AOI interaction was found for both number of fixations (*p* < 0.001,) and fixation duration (*p* < 0.001). Elite athletes allocated a significantly higher proportion of their fixations (54.3%) and viewing time (59.8%) to the Target, while minimizing attention to Other Areas (14.7% of fixations, 6.3% of duration). Novices displayed a dispersed attentional pattern, with more fixations on non-essential areas. Elites also demonstrated greater cognitive economy, evidenced by a lower total number of fixations and shorter total fixation duration than novices. Critically, fixation measures on the Target were positively correlated with performance scores (strongest in elites: *r* = 0.53, *p* < 0.01), whereas attention to Other Areas and the Stone Path was negatively correlated.

**Conclusion:**

Elite performance in wheelchair curling is characterized by highly selective and efficient visual attention. Experts excel not only in focusing on task-critical information but also in actively suppressing distractions, a hallmark of automated cognitive processing. These findings extend the “Quiet Eye” and cognitive efficiency theories to wheelchair curling, highlighting the critical role of perceptual-cognitive skills. They provide a robust empirical basis for developing targeted attention training protocols to accelerate expertise development in precision sports for athletes with disabilities.

## 1 Introduction

Wheelchair curling, a sport of precision and strategy within the Paralympic movement, presents a unique context to study the interplay of perception, cognition, and action under physical constraints (Laschowski et al., 2017; Li et al., 2022; World Curling Federation, 2025). The seated delivery position fundamentally alters the visual perspective and the process of gathering environmental information compared to standing athletes, placing a premium on efficient visual-attentional control as a cornerstone of expertise (Howard, 2010; Wang et al., 2022; Hua et al., 2025).

Eye-tracking research in sports sciences has consistently revealed that expert athletes possess a refined perceptual-cognitive architecture (Mann et al., 2007; Pojskic et al., 2020). They exhibit more economical visual search patterns, characterized by fewer but longer fixations on critical locations, culminating in the “Quiet Eye” (QE)—a final prolonged fixation on a relevant target before movement initiation, associated with optimal motor planning and performance under pressure (Vickers, 2007; González et al., 2017; Mizusaki et al., 2025). These superior gaze behaviors are theorized to reflect highly developed mental representations and cognitive processing efficiency, honed through years of deliberate practice (Ericsson and Lehmann, 1996; Zacharias et al., 2024).

Similar expertise-related visual strategies have been observed across various precision and aiming sports. For instance, in rifle shooting, experts exhibit longer and more stable QE periods compared to novices (Janelle et al., 2000; Williams et al., 2002). In air pistol shooting, elite performers demonstrate more efficient gaze behavior and attentional control linked to superior outcomes (Güler et al., 2023; Bayram et al., 2024). Furthermore, expertise in such tasks is also associated with enhanced executive functions, underscoring the intertwined nature of perceptual and cognitive processes (Shao et al., 2020).

Despite the acknowledged role of vision in curling strategy and execution (Bradley, 2009; Millet et al., 2021), scientific inquiry into the visual attention of athletes in this domain, particularly within the context of Paralympic sports, is scarce. Preliminary studies on able-bodied curlers suggest they employ specific visual strategies for “reading the ice” (Li et al., 2024; Song et al., 2022). Furthermore, studies in other team sports like soccer have highlighted how perceptual-cognitive processes, including visual search behavior, are affected by factors such as physiological demands and game contexts (Casanova et al., 2013, 2022), and how attentional effort varies across different player positions during real match play (Ballet et al., 2023). However, a significant gap exists in understanding the visual attention characteristics of elite wheelchair curling athletes and the underlying cognitive mechanisms that facilitate their superior performance. Understanding these mechanisms is not only of theoretical interest for expertise research but also holds profound practical value for designing evidence-based perceptual-cognitive training interventions for athletes with disabilities.

Therefore, this study aimed to bridge this gap by systematically investigating the visual attention characteristics of wheelchair curling athletes across a spectrum of expertise using portable eye-tracking. We recruited a unique cohort, including Paralympic and World Championship gold medalists, and compared them with national and provincial champions. The objectives were: (1) to compare the differences in the number of fixations, fixation duration, and their spatial distribution among elite, general, and novice athletes; and (2) to analyze the relationship between these visual attention metrics and sports performance. Grounded in theories of expert performance and attentional control, we hypothesized that elite athletes would exhibit more selective and efficient visual attention patterns, characterized by a dominant focus on the target and effective suppression of irrelevant areas, and that these specific patterns would be most strongly correlated with superior performance scores.

## 2 Materials and methods

### 2.1 Participants

An *a priori* power analysis was conducted using G*Power 3.1.9.7 (Faul et al., 2007) for a one-way ANOVA with three groups. With an effect size *f* = 0.25, *α* = 0.05, and power (1-β) = 0.80 (Petway et al., 2020; López-Laval et al., 2016), the minimum required sample size was 42. We recruited 48 athletes to account for potential data attrition.

Forty-eight wheelchair curling athletes (24 male, 24 female; mean age = 28.6 ± 6.3 years) from the Heilongjiang Provincial Disabled Persons’ Federation participated. Participants had various physical disabilities eligible for Paralympic wheelchair curling classification, primarily including spinal cord injuries and lower limb amputations. All participants provided written informed consent. Based on competitive achievement, participants were divided into three groups:

Elite Athletes (*n* = 16): Paralympic and World Championship gold medalists (e.g., 2018 PyeongChang, 2022 Beijing, 2023 & 2025 World Championships). Mean training experience: 11.4 ± 3.2 years.
General Athletes (*n* = 16): Gold medalists of the National Games of China. Mean training experience: 7.2 ± 2.5 years.
Novice Athletes (*n* = 16): Gold medalists of provincial-level games in China. Mean training experience: 3.8 ± 1.7 years.

All participants had normal or corrected-to-normal vision and no diagnosed visual perception disorders.

### 2.2 Apparatus and AOI definition

**Figure 1:**
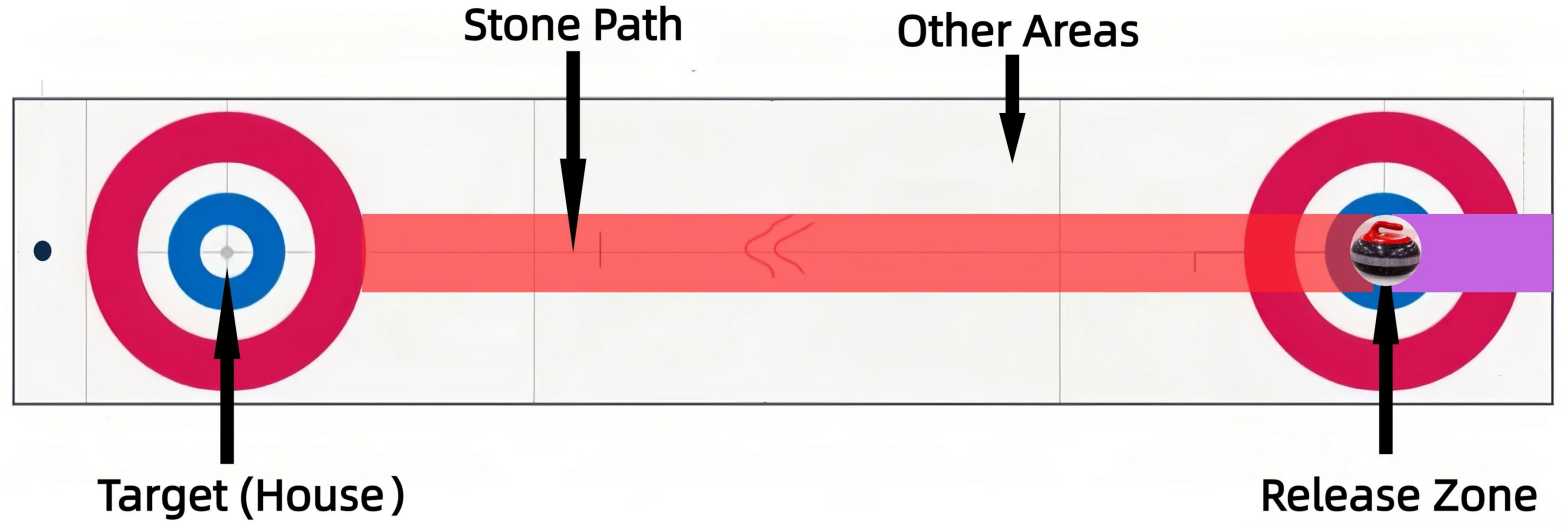
Defined areas of interest (AOIs) for fixation analysis in curling.

Visual attention data were collected using the Tobii Pro Glasses 3 wireless portable eye-tracker (Tobii, Sweden). The device has a sampling rate of 100 Hz and allows for natural movement during the actual delivery. The eye-tracker was calibrated for each participant according to the manufacturer’s guidelines prior to testing (Zhao et al., 2024). The visual field was segmented into four dynamic Areas of Interest (AOIs) for analysis (Figure 1). These AOIs were selected based on the fundamental phases of the curling delivery and key visual information sources identified in prior curling research (Bradley, 2009; Li et al., 2024):

Release Zone: The immediate area of stone release (critical for initial motor control).
Stone Path: The anticipated trajectory from the hack to the house (relevant for trajectory prediction and online adjustment).
Target (House): The circular scoring area (the ultimate goal for action planning).
Other Areas: Fixations outside predefined AOIs, indicating potential distraction.
  (AOIs were manually defined by a primary researcher and subsequently verified by a second independent researcher to ensure consistency).

### 2.3 Experimental design and procedure

A between-groups design was employed. The experiment was conducted on a standard international wheelchair curling sheet under competition-standard ice conditions (−5 °C ± 0.5 °C) at the Heilongjiang Curling Training Center.

The experiment was conducted at the Heilongjiang Curling Training Center under experimenter supervision from March 6 to March 9, 2024. Prior to formal testing, participants completed a standardized warm-up. The warm-up protocol consisted of 5 min of light upper-body mobility exercises (arm circles, shoulder rolls) followed by 5 practice deliveries with a stationary stone to re-familiarize with the delivery motion without performance pressure. The formal delivery sequence was randomized to control for order effects. Before testing, participants were fitted with the eye tracker, which was calibrated according to manufacturer specifications. Once successful calibration was confirmed, data recording commenced under the supervision of the equipment operator.

Performance was designed to simulate competitive conditions, assessing participants’ ability to accurately deliver stones to specific target zones. The target (a specific circle within the house) was verbally communicated to the athlete before each delivery attempt. The procedure adhered to official competition rules, with all deliveries initiated from the starting line. Each participant was permitted two practice throws followed by three consecutive formal delivery attempts using the same stone, delivered in a clockwise direction. Data from the three formal trials were averaged for each participant for statistical analysis. Sweeping was prohibited throughout the testing session (Li et al., 2024; Yamamoto et al., 2018).

**Figure 2:**
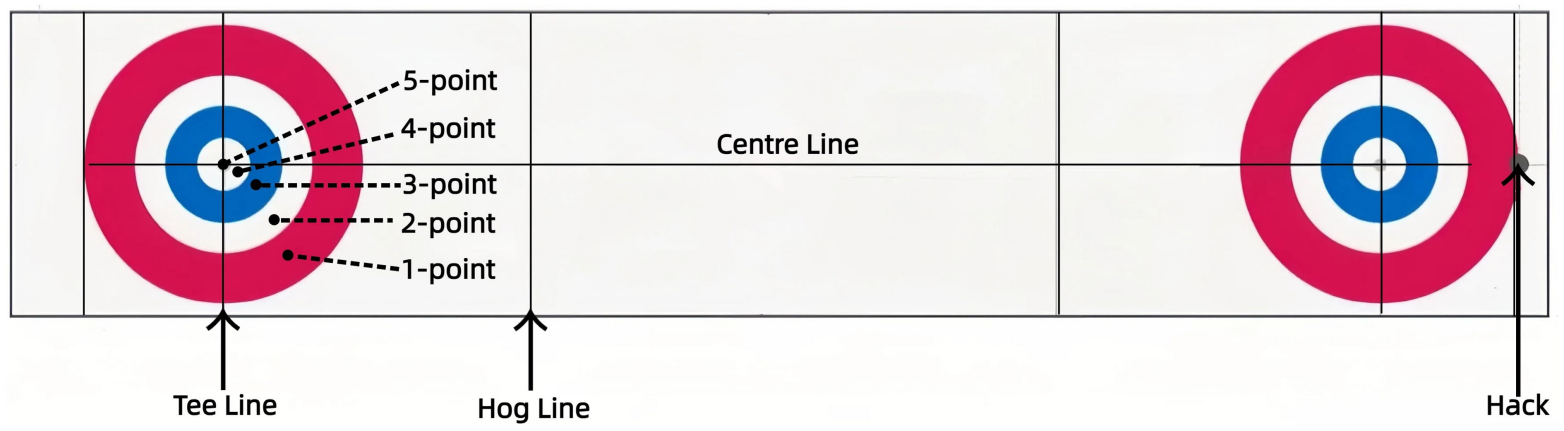
Curling rink and scoring zones in the curling performance.

Performance was evaluated by five internationally-certified judges, blinded to participant identity and sequence. Scoring was based on the final stone position relative to the house’s concentric circles (Figure 2): 5 points for the button (center circle), 4 for the one-foot ring, 3 for the four-foot ring, 2 for the eight-foot ring, and 1 for the twelve-foot ring. Stones on a boundary were awarded the higher score (Li et al., 2024; Yamamoto et al., 2018).

### 2.4 Data analysis

Eye-tracking data were processed in Tobii Pro Lab (v1.19) using the I-VT fixation filter (velocity threshold: 30°/s; minimum duration: 160 ms). For each trial, we extracted the number of fixations, fixation duration, and their distribution across AOIs. These specific gaze metrics—number of fixations and fixation duration—are widely used in sports eye-tracking research to quantify visual search efficiency and attentional allocation (Casanova et al., 2022).

Statistical analyses were performed in SPSS 26.0. After confirming normality and homogeneity of variance, two-way mixed ANOVAs (Group × AOI) were conducted for number of fixations and fixation duration, with AOI as the within-subjects factor and Group (Elite, General, Novice) as the between-subjects factor. One-way ANOVAs assessed group differences in total fixation metrics. Post-hoc tests used Bonferroni corrections. Pearson correlation analyses were performed separately for each group to examine the relationship between AOI-specific metrics and performance scores. Significance was set at *p* < 0.05. Effect sizes are reported as partial eta-squared ( $\eta_{p}^{2}$ ).

## 3 Results

### 3.1 Number of fixations across AOIs

A two-way mixed ANOVA revealed significant main effects of Group (*F*_(2,150)_ = 8.65, *p* < 0.001, $\eta_{p}^{2}$ = 0.12) and AOI (*F*_(3,150)_ = 282.15, *p* < 0.001, $\eta_{p}^{2}$ = 0.86), and a significant Group × AOI interaction (*F*_(6,150)_ = 5.17, *p* < 0.001, $\eta_{p}^{2}$ = 0.17). Post-hoc tests showed that elite athletes made significantly fewer fixations on “Other Areas” than both general (*p* = 0.028) and novice athletes (*p* < 0.001). No other significant between-group differences were found for specific AOIs (Table 1).

**Table 1:** Descriptive statistics and group comparisons for the number of fixations across AOIs (unit: times).

AOI	Elite	General	Novice	*F*	*p*	Partial *η^2^*
Release zone	4.31 ± 0.79	4.63 ± 0.89	5.44 ± 1.21			
Stone path	5.19 ± 1.33	5.94 ± 1.06	6.31 ± 1.08			
Target (House)	16.38 ± 1.96	15.31 ± 1.40	14.56 ± 1.75			
Other areas	4.44 ± 1.41	6.69 ± 1.20	8.06 ± 1.06			
Main effect: group				8.65	** < 0.001	0.115
Main effect: AOI				282.15	** < 0.001	0.862
Interaction: group × AOI				5.17	** < 0.001	0.171

** < 0.001.

### 3.2 Fixation duration across AOIs

A two-way mixed ANOVA revealed significant main effects of Group (*F*_(2,150)_ = 25.71, *p* < 0.001, $\eta_{p}^{2}$ = 0.26) and AOI (*F*_(3,150)_ = 1442.10, *p* < 0.001, $\eta_{p}^{2}$ = 0.97), and a significant Group × AOI interaction (*F*_(6,150)_ = 24.71, *p* < 0.001, $\eta_{p}^{2}$ = 0.50). Post-hoc tests revealed a clear expertise gradient (Table 2): elite athletes had significantly longer fixation durations on the Target than novices, and on the Release Zone than novices. Conversely, novices exhibited significantly longer fixation durations on the Stone Path and Other Areas than both elite and general athletes. General athletes also fixated longer on the Stone Path and Other Areas than elite athletes.

**Table 2:** Descriptive statistics and group comparisons for the fixation duration across AOIs (unit: ms).

AOI	Elite	General	Novice	*F*	*p*	Partial *η^2^*
Release zone	6,646 ± 344	6,093 ± 367	5,423 ± 346			
Stone path	3,082 ± 592	3,728 ± 521	5,100 ± 682			
Target (house)	16,956 ± 1,377	16,256 ± 629	15,531 ± 1,191			
Other areas	1883 ± 571	3,648 ± 483	4,937 ± 1,295			
Main effect: group				25.71	** < 0.001	0.255
Main effect: AOI				1442.10	** < 0.001	0.966
Interaction: group × AOI				24.71	** < 0.001	0.497

** < 0.001.

### 3.3 Total visual attention metrics and distribution

One-way ANOVAs revealed significant group effects on the total number of fixations (*F* (2,45) = 6.24, *p* = 0.004, $\eta_{p}^{2}$ = 0.21) and total fixation duration (*F*_(2,45)_ = 4.56, *p* = 0.016, $\eta_{p}^{2}$ = 0.17). Novice athletes had a significantly higher total number of fixations than elites (*p* = 0.003), and a longer total fixation duration than elites (*p* = 0.013) (Table 3).

**Table 3:** Descriptive statistics and group comparisons for the total visual attention metrics.

Dependent variable	Group	Mean ± SD	*F*	*p*	Partial *η^2^*
Total number of fixations (times)	Elite	30.19 ± 2.26	6.24	**0.004	0.214
	General	32.31 ± 1.74			
	Novice	34.13 ± 1.82			
Total fixation duration (ms)	Elite	28,438 ± 1,440	4.56	*0.016	0.166
	General	29,715 ± 673			
	Novice	30,976 ± 1,560			

* < 0.05, ** < 0.01.

**Figure 3:**
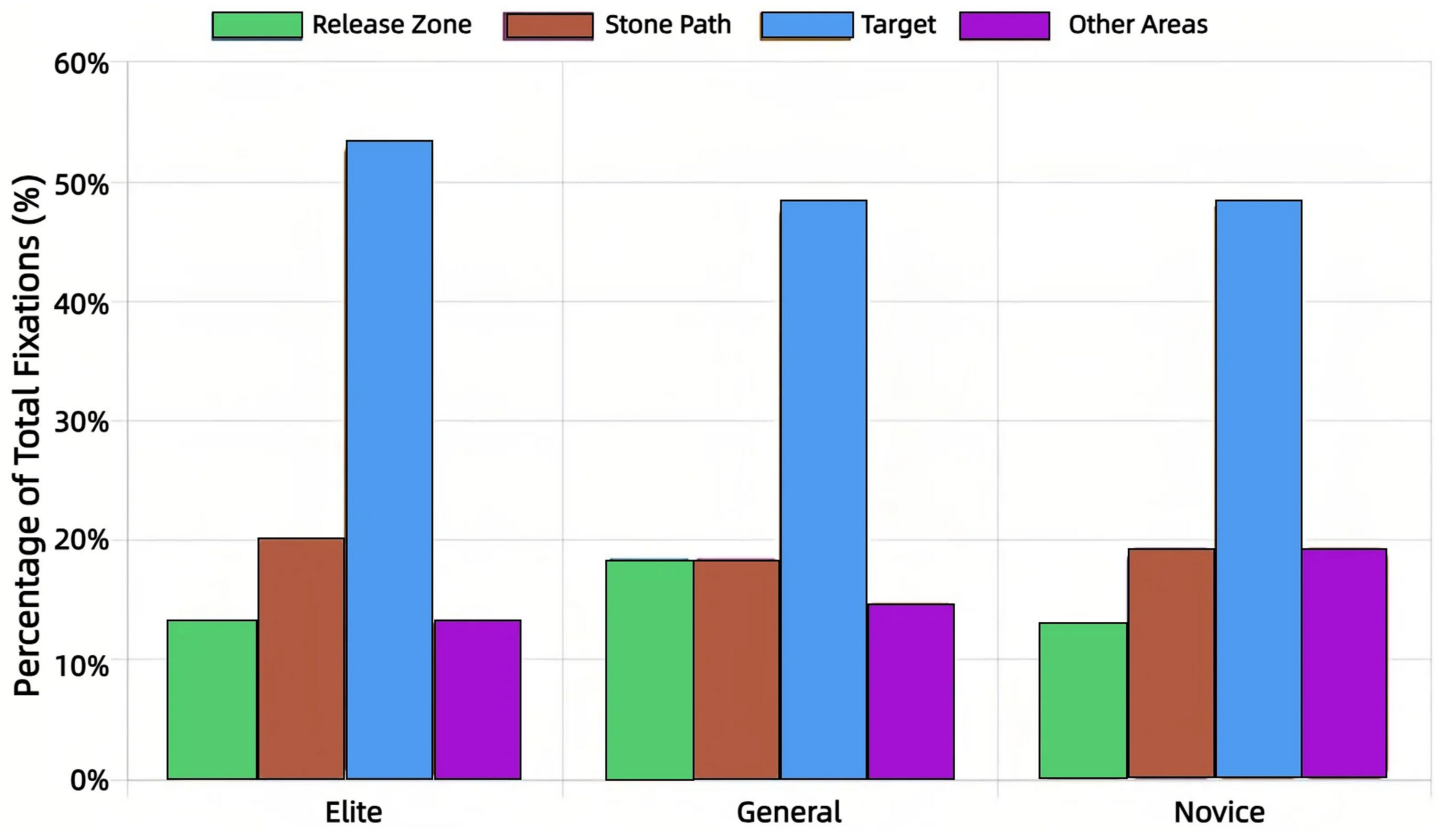
Distribution of number of fixations across AOIs by expertise level.

**Figure 4:**
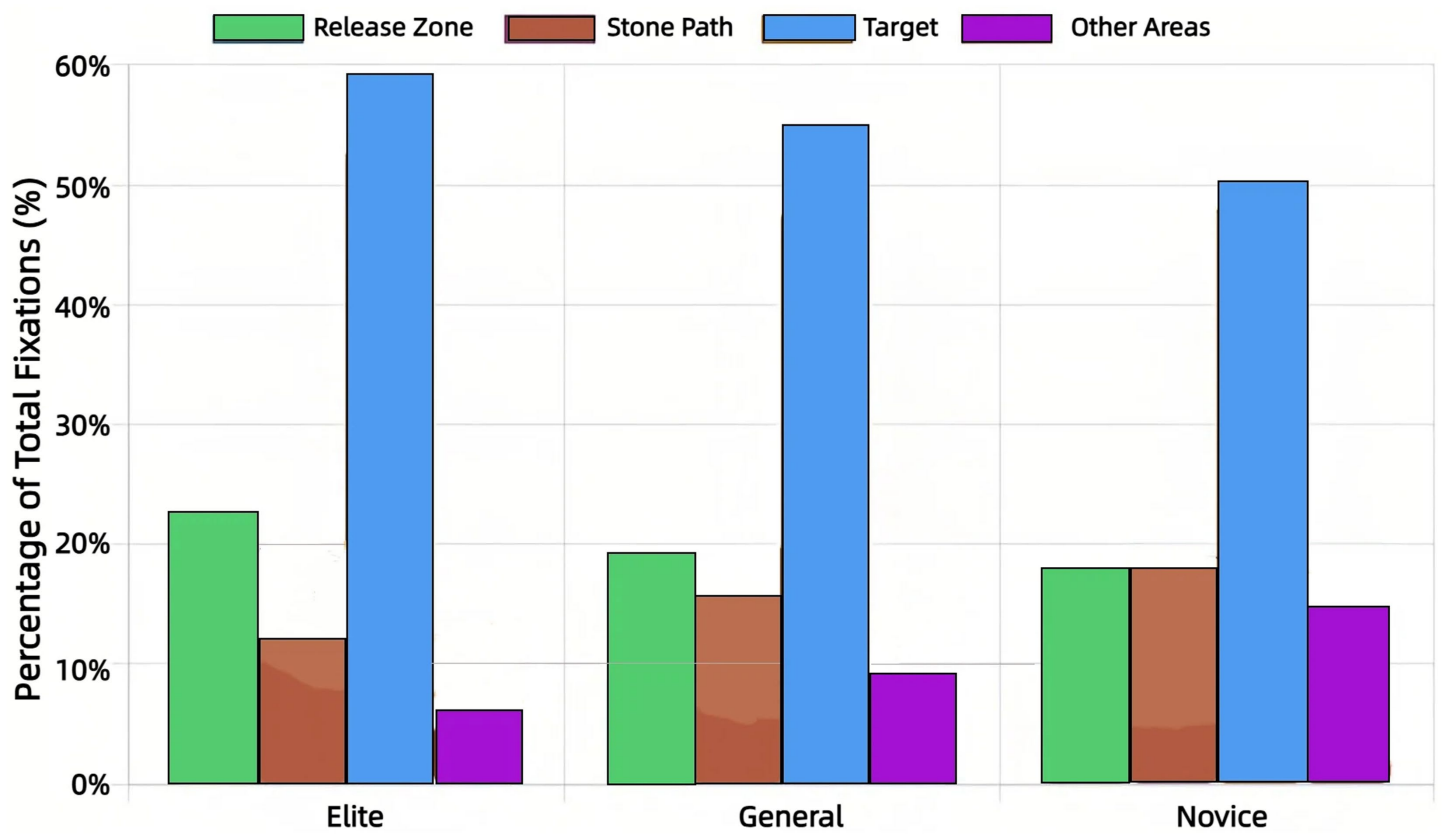
Distribution of fixation duration across AOIs by expertise level.

The distribution of visual attention (Figures 3, 4) highlighted stark expertise differences. Elite athletes allocated the majority of fixations (54.3%) and viewing time (59.8%) to the Target, with minimal allocation to Other Areas (14.7% of fixations, 6.3% of duration). Novices showed a dispersed pattern (Target: 42.6% of fixations, 49.2% of duration; Other Areas: 25.1% of fixations, 16.1% of duration). General athletes displayed an intermediate profile.

### 3.4 Relationship between fixation patterns and performance scores

**Figure 5:**
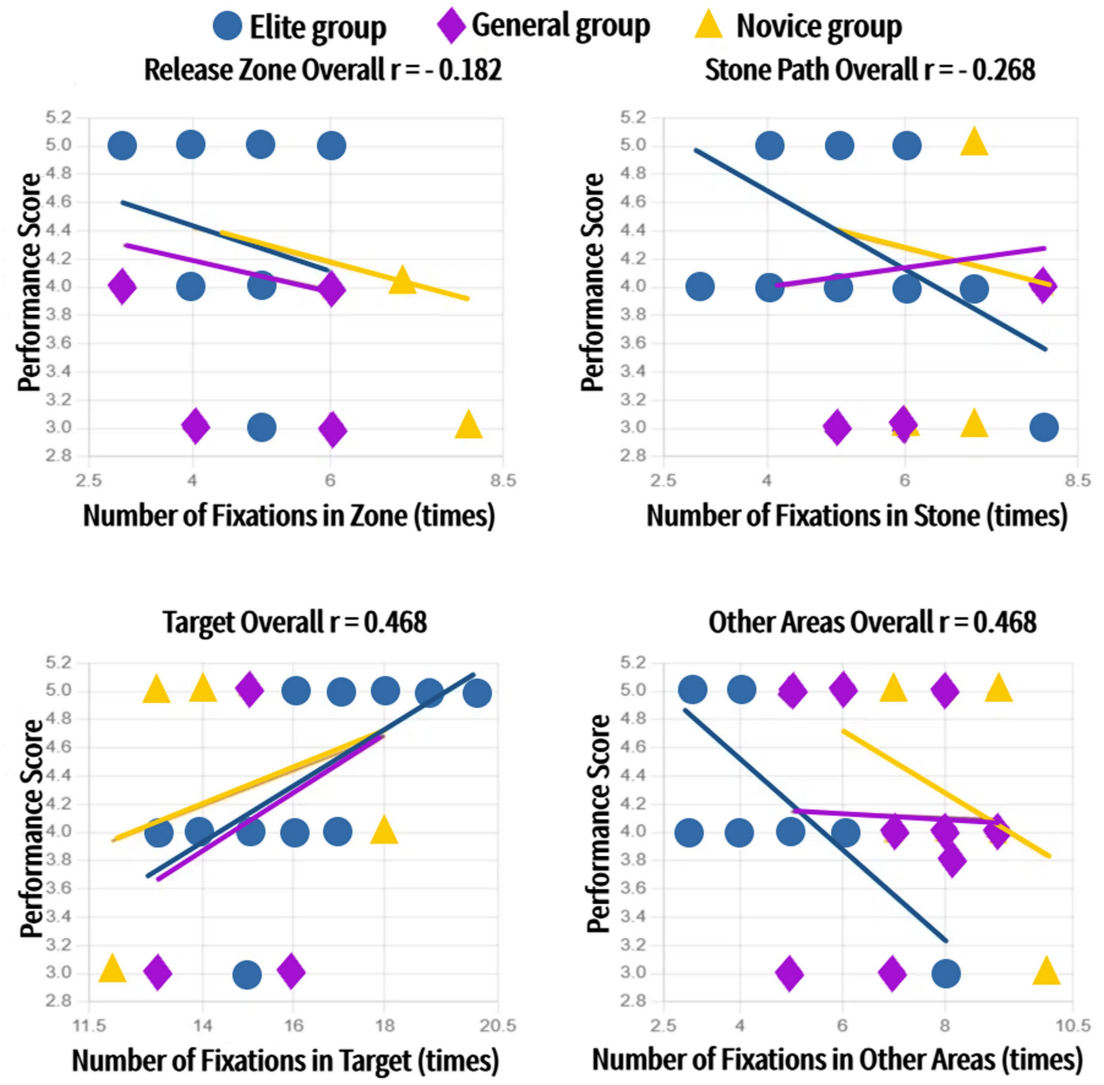
Relationships between fixation counts in different AOIs and performance scores for each group (Elite, General, Novice).

**Figure 6:**
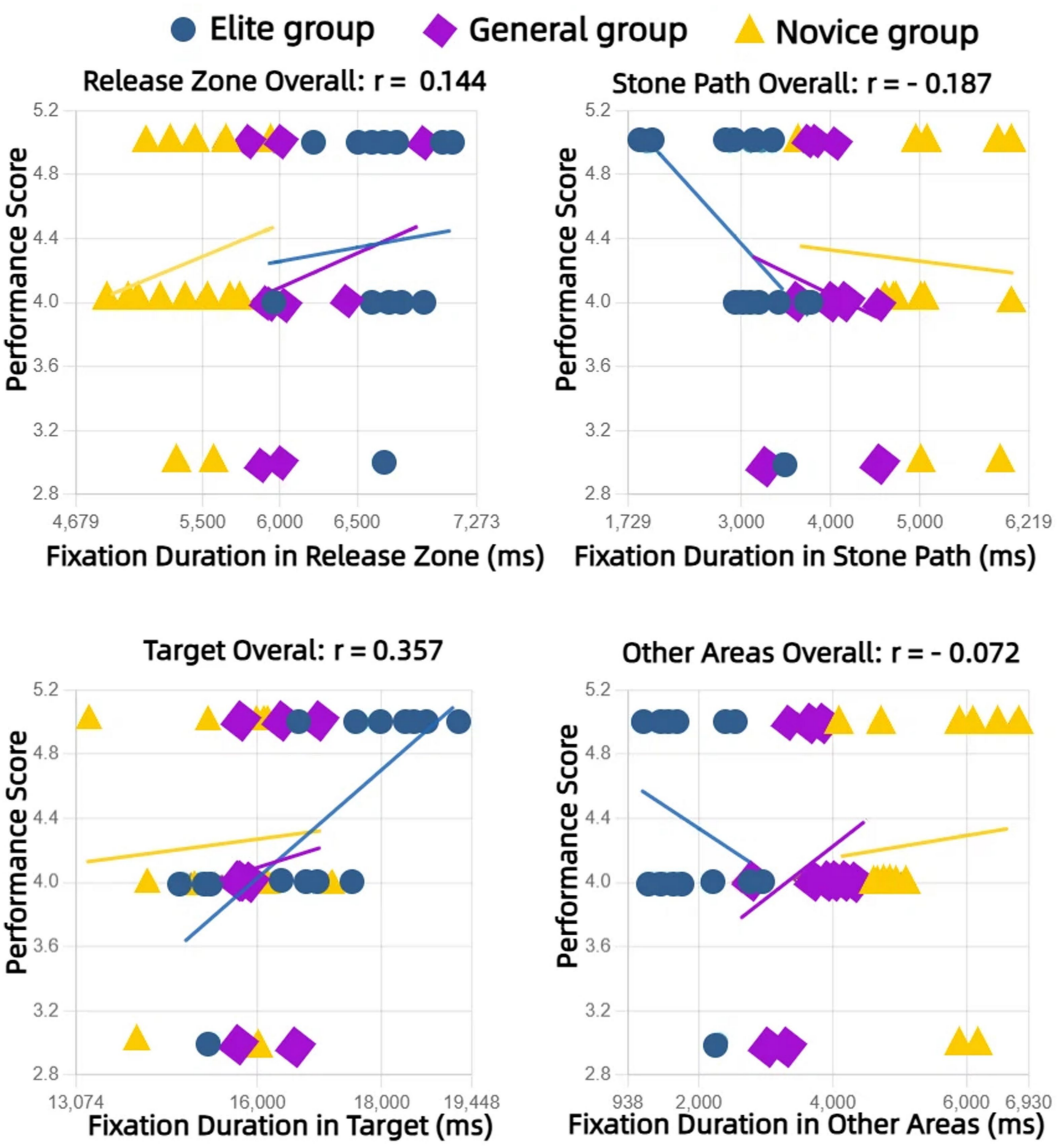
Relationships between fixation duration in different AOIs and performance scores for each group (Elite, General, Novice). Figures 5, 6 have been revised to improve readability by increasing font sizes and reducing visual clutter, while Figure 6 is presented here as an example of the improved format.

Separate correlation analyses for each group revealed that number of fixations and fixation duration on the Target were positively correlated with performance scores (strongest in elites: *r* = 0.53, *p* < 0.01 for number of fixations; *r* = 0.50, *p* < 0.01 for fixation duration). Conversely, fixations on Other Areas (strongest negative in elites: *r* = −0.48, *p* < 0.01 for number of fixations; *r* = − 0.46, *p* < 0.01 for fixation duration) and the Stone Path were negatively correlated with performance (Figures 5, 6).

## 4 Discussion

This study provides a novel investigation into the cognitive mechanisms of visual attention in wheelchair curling athletes, revealing systematic expertise-related differences that align with and extend established theories of perceptual-cognitive expertise.

Our central finding is the superior attentional selectivity and cognitive efficiency of elite athletes. Elites allocated over half of their visual resources (number of fixations and fixation duration) to the task-critical Target area, while simultaneously exhibiting the most effective suppression of irrelevant information, as evidenced by minimal attention to Other Areas. This pattern signifies a highly refined “knowledge-driven” attentional control system (Vickers, 2007; Klatt and Smeeton, 2022), where cognitive resources are deployed based on the paramount requirements of the task. The significant Group × AOI interaction underscores that expertise is defined not by a general increase in looking, but by a qualitative shift in what is attended to and what is ignored. This finding resonates with the Attentional Control Theory (Eysenck et al., 2007), which posits that anxiety and expertise influence the balance between goal-directed (top-down) and stimulus-driven (bottom-up) attentional systems. The elite athletes’ pattern suggests a stronger top-down control, efficiently prioritizing task-relevant cues while inhibiting distractors.

The fixation duration patterns offer strong support for the application of the Quiet Eye (QE) theory to wheelchair curling. The elites’ prolonged fixations on the Target and Release Zone are indicative of an extended QE period, facilitating optimal motor parameterization and online control during critical movement phases (Vickers, 2007; González et al., 2017). Conversely, the novices’ longer dwell time on the Stone Path suggests a “reactive” or “feedback-dependent” strategy, potentially attempting to track an anticipated path rather than trusting a pre-programmed action—a hallmark of less automated skill (Mann et al., 2007; Babiloni et al., 2009).

Furthermore, the finding that elite athletes achieved superior performance with a lower total number of fixations and shorter total fixation duration is a clear marker of cognitive economy. This efficiency suggests that experts extract the necessary information more rapidly and with fewer samples, relying on rich internal models built through extensive practice (Presta et al., 2021; Ericsson and Lehmann, 1996). Novices, with less refined cognitive representations, appear to engage in more effortful and extensive visual searching, implying a less efficient and more capacity-consuming processing style (Slagter et al., 2018; Burris et al., 2020).

The correlation results powerfully link these gaze behaviors to performance outcomes. The strong positive correlation between Target-looking and performance, most pronounced in elites, confirms the functional value of this focus. More critically, the strong negative correlations for Other Areas and the Stone Path in elites indicate that for experts, the ability to inhibit distracting or non-essential information is as crucial as the ability to focus (Hülsdünker et al., 2018; Harrison et al., 2023). This highlights a dual mechanism of expert attention: enhanced facilitation of relevant stimuli coupled with active suppression of irrelevant stimuli (Cui et al., 2025; Pusenjak et al., 2015). This finding moves beyond simply identifying “where experts look” to explaining “how” their cognitive system achieves superior performance through efficient resource management.

An alternative explanation for the observed efficiency could be attributed to greater task familiarity and movement automatization among elites, which inherently reduces the need for conscious visual monitoring of the action itself (Stone Path) or the environment (Other Areas). While our design cannot fully disentangle automatization from active inhibition, the strong negative correlations specifically for elites suggest that superior performance is associated not just with less looking elsewhere, but with an active, strategic avoidance of non-critical information—a skill that likely develops alongside automatization.

From an applied perspective, these findings have direct implications for training in Paralympic sports. Developing perceptual-cognitive training protocols for wheelchair curling athletes should emphasize cultivating both selective focus (e.g., through QE training) and distraction inhibition. This could involve gaze-contingent feedback systems that alert athletes when their focus drifts from the target, or simulated training environments with controlled visual distractors. Such interventions would be particularly valuable for athletes with disabilities, as they offer a pathway to enhance performance that complements physical training and adapts to individual functional profiles.

## 5 Conclusion

This study demonstrates that expertise in wheelchair curling is underpinned by distinct and highly efficient visual-attentional strategies. Elite athletes exhibit a sophisticated cognitive profile characterized by:

- Superior Attentional Selectivity: A dominant focus on the task-critical target area (House).
- Effective Cognitive Inhibition: The ability to actively suppress attention to irrelevant and distracting areas.
- Enhanced Cognitive Economy: Achieving superior performance with less overall visual sampling, indicative of automated processing.

These findings robustly extend theories of attentional control and the Quiet Eye to the domain of precision Paralympic sports. They also suggest potential applicability of similar perceptual-cognitive training principles to other precision Paralympic disciplines, such as shooting or archery. They underscore that perceptual-cognitive skills are a critical component of expertise in wheelchair curling. Consequently, training interventions should be designed to cultivate not just the physical skill of delivery, but also the cognitive skills of focused attention and distraction inhibition, potentially through gaze-contingent feedback and video-based simulation training.

## 6 Limitations and future research

While this study was conducted in an ecologically valid setting, some limitations warrant mention. The sample was drawn from a single province; future multi-regional studies would enhance generalizability. Secondly, incorporating neurophysiological measures like EEG or fNIRS could directly probe the neural mechanisms of the observed attentional control and inhibition. Third, this study was conducted in a controlled environment; future research should examine how these fixation patterns hold under the heightened psychological pressure of actual competition. It is also important to note that eye-tracking captures overt attention (where one is looking), but not necessarily covert attention (where one is attending mentally without moving the eyes). Finally, a primary applied direction is the development and rigorous testing of perceptual-cognitive training protocols, based on these findings, to enhance both the visual-attentional skills and on-ice performance of developing athletes. Future longitudinal intervention studies are needed to establish a causal link between training-induced changes in gaze behavior and performance improvements.

## Data Availability

The original contributions presented in the study are included in the article/supplementary material, further inquiries can be directed to the corresponding author.
